# Relative Validity and Reproducibility of a New 44-Item Diet and Food Frequency Questionnaire Among Adults: Online Assessment

**DOI:** 10.2196/jmir.9113

**Published:** 2018-07-05

**Authors:** Aurélie Affret, Douae El Fatouhi, Courtney Dow, Emmanuelle Correia, Marie-Christine Boutron-Ruault, Guy Fagherazzi

**Affiliations:** ^1^ Inserm U1018 Center for Research in Epidemiology and Population Health Villejuif France; ^2^ Université Paris-Sud Orsay France; ^3^ Université de Versailles-Saint-Quentin-en-Yvelines Versailles France

**Keywords:** Short Food Frequency e-Questionnaire, Web-based, validity, reproducibility, online dietary assessment tool

## Abstract

**Background:**

Dietary questionnaires currently available which can assess the habitual diet are timely, costly, or not adapted well to the modern diet; thus, there is a need for a shorter food frequency e-Questionnaire (FFeQ) adapted to Western diets, in order to properly estimate energy and macronutrient intakes or rank individuals according to food and nutrient intakes.

**Objective:**

The aim of this study was to evaluate the relative validity and reproducibility of a 30-minute and 44-item FFeQ in a sample of adults obtained from the general population.

**Methods:**

A sample of French adults was recruited through social media and an advertising campaign. A total of 223 volunteers completed the FFeQ twice at one-year intervals and were included in the reproducibility study. During that interval, 92 participants completed three-to-six 24-hour recalls and were included in the validity study. Nutrient and dietary intakes were computed for all validity and reproducibility participants. The level of agreement between the two methods was evaluated for nutrient and food group intakes using classification into quintiles of daily intake, correlation coefficients and Bland-Altman plots.

**Results:**

For relative validity, correlation coefficients ranged from 0.09 to 0.88 (unadjusted correlation coefficients, median: 0.48) and 0.02 to 0.68 (deattenuated and energy adjusted correlation coefficients, median: 0.50) for food group and nutrient intakes, respectively. The median proportion of subjects classified into the same or adjacent quintile was 73% and 66% for food and nutrient intakes, respectively. Bland-Altman plots showed good agreement across the range of intakes. Regarding reproducibility, intraclass correlation coefficients ranged from 0.33 to 0.72 (median: 0.60) and 0.55 to 0.73 (median: 0.64), for food and nutrient intakes, respectively.

**Conclusions:**

The FFeQ showed acceptable validity and reproducibility in a sample of adults based on their food and nutrient intakes. The FFeQ is a promising and low-cost tool that can be used in large-scale online epidemiological studies or clinical routines and could be integrated into evidence-based smartphone apps for assessing diet components.

## Introduction

A healthy lifestyle, characterized by an adequate, balanced diet combined with regular physical activity, is one of the determinants for good health [1]. Moderate to strong associations between healthy dietary patterns and decreased risk of obesity and chronic diseases like cardiovascular disease, hypertension, type 2 diabetes, and some cancers are strongly highlighted in the literature [2,3].

Among the many available tools that evaluate individual dietary intakes, the food frequency questionnaire (FFQ) has been frequently used in nutritional epidemiology studies since the 1980s [4-6]. Despite limitations (difficulties to estimate habitual intakes, memory bias, errors in perception of portion sizes, and use of restricted food lists), FFQs collect valuable information allowing researchers to assess the typical diet at a low cost and logistic burden, and it can be self-administered [5-7]. FFQs also allow researchers to accurately rank subjects according to their dietary and nutritional intake, which is important when comparing risks in various subgroups [6].

As previously described [8], a consortium of six cohort studies (E3N [9], E4N [10], CKD-REIN [11], i-Share [12], Elfe [13] and Psy-COH) was established to create a unique Food Frequency Questionnaire (FFQ) that could quickly assess the habitual diet in several populations: adults, the elderly, adolescents, students, patients with mental disorders, and patients with chronic kidney disease. It was decided that the questionnaire would be limited to 50 items to quickly assess the diet.

Existing FFQs of 50 items or less have not been proven suitable to quickly and accurately assess the diet in several French population subgroups: i) the FFQ by Vercambre et al [14] was developed for senior women and focused on specific nutrients for that population, ii) one third of the items of the FFQ developed by Giovannelli et al [15] were not valid and a food composition table was not available to study nutritional intakes, iii) the FFQ developed by Barrat et al [16] referred to intakes during the preceding week, and did not consider seasonal variability, and iv) two FFQs were developed solely in the context of cardiovascular prevention [17,18]. Therefore, the consortium decided to develop a new and unique FFQ (40 items) adapted to several population subgroups of interest. They agreed that some additional, specific questions (10 items maximum, as performed in the present study) could be added to the questionnaire to help estimate specific nutrients of interest or to obtain qualitative information about the dietary context, consistent with the targeted populations. In a pilot study, a paper version of the consortium FFQ was validated in a sample of patients with chronic kidney disease [8] and showed acceptable validity and reproducibility. Then, a web version of the questionnaire was developed by the consortium to meet the need of a food frequency e-Questionnaire (FFeQ) adapted to the diet of those in Western countries, able to accurately estimate energy and macronutrient intakes, and to rank individuals according to food and nutrient intakes.

Web-based dietary assessments provide a lot of advantages [19,20]: they have the potential to save time and financial resources, may be preferred by participants, and response quality can be improved directly by including cutoff values and alert messages in case of inconsistencies, abnormal or missing data. Several examples of 24-hour recall and Web-based FFQs already exist in the literature [21-26]. Because 24-hour recalls need to be repeated to assess the overall diet, Web-based FFQs might be more feasible in large-scale studies. However, most of the existing Web-based FFQs are long questionnaires and therefore time-consuming [21,24,25].

Before using a newly developed or modified FFQ, it must first be validated to be considered an acceptable method of dietary assessment [27]. The aim of the present study is to study the reproducibility of the online version of the newly developed FFQ (FFeQ) and evaluate its relative validity against 24-hour recalls in a sample of French adults.

## Methods

### Study Population and Design

According to Willett [6], the number of subjects necessary to conduct reproducibility and validity studies is approximately 110. Between January and February 2016, a national invitation to participate in the present reproducibility and validity study was advertised in the Inserm (*French* National *Institute* of Health and Medical Research) network and through the Inserm’s Twitter and Facebook accounts. In total, 441 adults volunteered (from which 214 participants agreed to participate in the validity study), provided their informed consents, and were invited to complete the FFeQ. They were asked to complete the FFeQ twice, at a one-year interval, in February-April 2016 and again in February-April 2017. In total, 319 participants completed the FFeQ once (the 122 participants who failed to complete the first questionnaire were more likely to be women and live in the South and East of France and overseas). Two hundred twenty-nine participants completed it twice (participants who completed both questionnaires were more likely to be women and live in the South of France). Participants who under- or over- reported energy intake in one of the FFeQs, ie, were in the top and bottom 1% of the energy intake-to-energy requirement ratio distribution, were excluded as previously described [28]. Energy requirement was calculated as follows: Basal Metabolic Rate (BMR)* Physical Activity Level (the cutoff value of 1.55 for a minimal activity level was chosen [29]). BMR was computed based on sex, age, height, and weight, using the Schofield formula [30]. After exclusion, a total of 223 participants were included in the reproducibility study. Among them, 92 patients answered at least three (out of six) 24-hour recalls and were therefore included in the relative validity study (a flow diagram is presented in Multimedia Appendix 1). We decided to include participants with at least three recalls in the validity study to ensure i) reasonable intra-individual variations, ii) seasonal representation and iii) sufficient statistical power. Participants who completed at least three 24-hour recalls were more likely to be older than participants who completed the first questionnaire but did not complete three 24-hour recalls. They had higher energy intake and healthier dietary habits than their counterparts (data not tabulated).

From the 214 participants who volunteered for the validity study, only 130 were interviewed for the 24-hour recalls. The main reasons were contact difficulties as they were mostly active people, and that this study was conducted by only one dietitian which prevented us from interviewing several participants simultaneously.

### Food Frequency Questionnaire

The food list for the FFeQ was developed by the investigators based on existing national food questionnaires [14-16,31-34] and data from the second national study of individual food intakes of French adults [35] to ensure that all food groups contributing to at least 5% of the mean energy, macronutrient, vitamin, or mineral intake of the French population were represented in the FFeQ, [16]. The FFeQ was self-administered online. The questionnaire asked participants to report their usual dietary intake over the past year. In epidemiological studies, one-year memory FFQ are mainly used because they assess long-term diets (diets tend to remain stable year on year) and season variability of intakes is considered [6].

The questionnaire was divided into two parts: The first part comprised 40 food groups. It quantified consumption by frequency (never or less than once a month, *x* times a day, *x* times a week or *x* times a month) and portion sizes per food group item. Photos previously validated [36] were directly integrated into the questionnaire to help participants estimate the consumed quantities of 21 food items (see Multimedia Appendices 2 and 3). Most of the time, there were three photos showing increasing portion sizes with five possible answers (less than the lowest portion, the lowest portion, an intermediate portion, the biggest portion, more than the biggest portion). For items not having a photo, participants were asked to quantify their consumption based on a standard portion size (typical household measurements like measuring spoons or standard units such as individual containers of yogurt).

The second part was specific to the study population. It was composed of 10 questions. Six were qualitative questions about eating habits (eg, meal frequency, socialization during meals, source of food supplies), and four questions were used to obtain nutritional data, of which two provided more detailed information about some food groups from the first part of the questionnaire (ie, fish and soft drinks).

In total, 44 items were used to obtain the nutritional data (see Multimedia Appendix 4). Daily intakes for each food group item were computed: frequencies were converted into numbers of servings per day and multiplied by the portion size. An ad hoc composition table was developed using data from the INCA2 French representative population survey (35) to estimate the percentage of contribution of each food included in a food group item. Nutritional data were then obtained using the French food composition database established by the French Data Centre on Food Quality (Ciqual, last updated in 2013) [37]. Besides nutritional and diet context information, information on sex, birth date, and anthropometric data was elicited. It also questioned participants about potential changes in their food habits during the past year due to specific situations (diet, pregnancy, a move, surgery or depression).

The FFeQ was adapted for laptops, tablets and smartphones. Compared to the paper version of the questionnaire [8], the online version presented here had a higher data quality thanks to alerts, restricted answers and automated checks. The design of the FFeQ consisted of one web page per food item and a pilot study previously demonstrated an average time of 30 minutes to complete the questionnaire.

### 24-Hour Recalls

The reference method used to compare results from the FFeQ consisted of six 24-hour recalls carried out every two months during the year between the first and the second FFeQ. Study participants were asked to recall all foods and beverages consumed on the previous day (due to logistics, data for Saturdays were collected on Mondays). Participants were not informed in advance of the day of the recall. To account for intra-individual variation (because dietary habits may differ according to weekdays or weekends and seasons), all days and all seasons were covered by the recalls as recommended [6] ie, days for recalls were randomly selected every two months per participant. Phone interviews were carried out by a trained dietitian who entered the data into the Nutrilog Software (v3.10b). These data were instantly converted into nutrient intakes by the software using the Ciqual food composition database [37]. A validated photo album of 42 foods [34] was previously e-mailed to the participants to help them quantify the amount of food consumed during the phone interview.

### Statistical Analysis

We computed descriptive statistics (median and interquartile range) for nutrients and foods for both FFeQs and the average of the 24-hour recalls. Wilcoxon signed rank tests were performed to assess whether the mean ranks differed between groups.

### Relative Validity

To study relative validity, data evaluated by the second FFeQ (FFeQ2) were compared with the mean of the 24-hour recalls, since both methods covered the same period. A list of concordance was established between food group items from the FFeQ and food items provided by 24-hour recalls. Few rarely consumed foods declared during the 24-hour recalls were not covered by the FFeQ items and were not taken into consideration.

Unadjusted Spearman correlation coefficients were calculated for food groups. Unadjusted and energy-adjusted Pearson correlation coefficients were calculated for nutrient intakes. Energy-adjusted coefficients, corrected for attenuation for within-person variation in the reference method (deattenuated coefficients) [6,38], were produced. Energy adjustment was performed using the residual method [6]. To improve the normal distribution, nutrient intakes were logarithmically transformed before analysis.

In terms of food group and nutrient intakes, we examined the level of agreement in ranking subjects between the two methods through cross-classification into quintiles. The percentage of participants classified in the lowest quintile in the FFeQ and the highest quintile in the 24-hour recalls (and vice versa) was studied. Because several food groups had a proportion of non-consumers >20%, we established three categories as follows: class=1 for null consumption; class=2 for consumption below or equal to the median value in consumers; class=3 for consumption over the median value in consumers. For food groups having a proportion of non-consumers <20%, subjects were classified into tertiles of consumption.

We evaluated agreement between the FFeQ and the 24-hour recalls performing Bland-Altman plots on energy-adjusted values [39-41]. Mean differences between the two assessment methods were plotted against the average estimation of the two methods. The 95% limit of agreement was calculated as the mean difference (SD 1.96).

### Reproducibility

To evaluate reproducibility, data obtained from the first and second FFeQs (FFeQ1 and FFeQ2) were compared. For food groups, unadjusted Spearman correlation coefficients and intraclass correlation coefficients (ICC) were estimated. Unadjusted and energy-adjusted Pearson correlation coefficients as well as ICC were calculated for nutrient intakes. Nutrient intakes were logarithmically transformed before analysis, to improve the normal distribution. The level of agreement in ranking subjects between the two FFeQs (in terms of food group and nutrient intakes) was examined through cross-classification into quintiles. All statistical analyses were performed on SAS 9.4 (SAS Institute Inc, Cary, NC, USA). A *P* value <.05 was considered statistically significant.

## Results

Baseline characteristics of the participants included in the relative validity and reproducibility studies are presented in Table 1. Participants included in both studies were mostly women (63.0%, 58 out of 92 participants and 74.9%, 67 out of 223 participants in the validity and reproducibility studies, respectively). Relative validity study participants were older than reproducibility study participants (47.7 years old, SD 14.9 vs 40.5 years old, SD 14.9) but the mean BMI was similar in both studies (23.5 kg/m^2^, SD 4.2 in the reproducibility study). Participants lived in all regions of France, with higher proportions living in Paris and suburbs, and in the South. Most of the participants included in the relative validity study had complete data for six 24-hour recalls (73.9%, 68 out of 92 participants).

### Relative Validity

Dietary intakes estimated by the FFeQ2 and the mean of the 24-hour recalls are presented in Table 2. Some food items such as “whole-grain pasta, rice, and wheat,” “legumes,” “milk” or “fruit” tended to be overestimated with the FFeQ whereas other food groups such as “raw vegetables,” “pizza, lasagna, and quiche,” “sausages and processed meat,” “cheese,” “sweet snacks, chocolate, and Danish pastries” or “alcoholic beverages excluding wine” were underestimated with the FFeQ2.

Unadjusted Spearman coefficients ranged from 0.09 (variety meats) to 0.88 (tea and herb teas), the median value being 0.48. Eight food groups had correlation coefficients below 0.3.

**Table 1:** Descriptive characteristics of the subjects included in the relative validity and reproducibility study.

Characteristic		Validity (n=92)	Reproducibility (n=223)
Sex (women), n (%)		58 (63.0)	167 (74.9)
Age (years), mean (SD)		47.7 (14.9)	40.5 (14.9)
Body mass index (kg/m^2^), mean (SD)		23.6 (3.3)	23.5 (4.2)
**Area of residence, n (%)**			
	South	15 (16.3)	60 (27.0)
	West	10 (10.9)	19 (8.6)
	North	1 (1.1)	8 (3.6)
	East	7 (7.6)	13 (5.9)
	Center	6 (6.5)	13 (5.9)
	Overseas departments	0 (0)	3 (1.4)
	Paris and suburbs	53 (57.6)	106 (47.7)
**Number of 24-hour recall days, n (%)**			
	3	5 (5.4)	N/A^a^
	4	4 (4.3)	N/A
	5	15 (16.3)	N/A
	6	68 (73.9)	N/A
**Distribution of 24-hour recall days (average %)**			
	Weekday	67 (73.3)	N/A
	Weekend	25 (26.7)	N/A
	Autumn and winter	49 (53.0)	N/A
	Spring and summer	43 (47.0)	N/A

^a^N/A: not applicable.

**Table 2:** Relative validity of the Food Frequency Questionnaire (FFeQ) for food groups (n=92). For food groups with a proportion of non-consumers >20%, tertiles and quintiles classifications were not performed. Instead, participants were classified as follows: class=1 for null consumption; class=2 for consumption below or equal to the median value in consumers, class=3 for consumption above the median value in consumers.

Food groups	Daily intakes^a^			FFeQ2 vs mean three to six 24-hour recalls			
	24-hour recalls, median (IQR)	FFeQ2, median (IQR)	FFeQ2-24-hour recalls, mean difference (SD)	Unadjusted Spearman correlation coefficients	Cross-classification of food group distribution, %		
					Subjects classified in same tertile	Subjects classified in same or adjacent quintile	Subjects classified in opposite quintiles
Whole-grain bread and substitutes^b^	18.7 (44.8)	10.7 (40.0)	-1.2 (54.1)	0.35	53	—^c^	—
White bread and substitutes	50.9 (57.7)	32.0 (69.3)	-2.2 (47.3)	0.63	54	78	1
Breakfast cereals^b^	0.0 (0.0)	0.0 (0.5)	1.9 (9.2)	0.68	79	—	—
Whole-grain pasta, rice and wheat^b,d^	0.0 (0.0)	2.3 (32.0)	24.2^d^ (46.4)	0.14^e^	49	—	—
White pasta, rice and wheat	68.3 (82.5)	45.3 (90.7)	-11.3 (68.2)	0.52	49	71	1
Legumes^b^	0.0 (16.7)	16.7 (20.0)	8.8^d^ (30.8)	0.26	27	—	—
French fries and other fried tubers^b^	0.0 (15.0)	8.7 (15.3)	2.3 (23.8)	0.35	40	—	—
Potatoes and other tubers (not fried)^b^	50.0 (64.6)	40.0 (53.3)	-4.8 (66.9)	0.26	45	—	—
Cooked vegetables	179.6 (163.0)	176.7 (166.7)	-8.8 (114.7)	0.49	50	68	1
Raw vegetables	63.1 (66.7)	34.4 (73.7)	-22.1^d^ (51.7)	0.53	58	74	1
Pizza, lasagna and quiche^b^	27.9 (58.6)	14.2 (15.0)	-23.2^d^ (40.3)	0.44	48	—	—
Sandwich, burgers and kebab^b^	0.0 (23.3)	0.0 (12.0)	-2.6 (18.2)	0.52	61	—	—
Fish fingers/breaded meat^b^	0.0 (0.0)	0.0 (3.3)	-4.1^d^ (14.2)	0.27	64	—	—
Sausages and other processed meat^b^	30.0 (30.3)	11.3 (28.0)	-14.9^d^ (29.5)	0.38	54	—	—
Poultry/rabbit^b^	24.2 (47.5)	20.0 (30.0)	4.1 (52.8)	0.45	41	—	—
Meat	43.3 (37.8)	26.3 (34.7)	-2.3 (44.4)	0.39	38	61	1
Variety meats^b^	0.0 (0.0)	0.0 (1.3)	1.7^d^ (10.0)	0.09^e^	74	—	—
Eggs^b^	8.3 (20.0)	14.1 (21.2)	6.5^d^ (26.8)	0.35	35	—	—
Fish^b^	15.8 (38.8)	13.3 (20.0)	-3.0 (26.2)	0.34	41	—	—
Seafood (excluding fish)^b^	0.0 (2.5)	0.0 (6.7)	-3.2 (19.1)	0.33	59	—	—
Milk^b^	0.0 (63.3)	0.0 (180.0)	70.6^d^ (175.2)	0.73	68	—	—
Yogurt, white cheese, cottage cheese	96.3 (109.8)	125.0 (123.3)	28.0^d^ (70.6)	0.75	60	88	0
Cream dessert^b^	0.0 (20.8)	2.0 (16.7)	5.1 (36.3)	0.40	53	—	—
Cheese	31.0 (30.6)	28.0 (18.0)	-7.1^d^ (25.2)	0.51	52	68	1
Butter, fresh cream	4.2 (6.7)	10.0 (20.0)	10.1^d^ (20.2)	0.66	51	79	0
Margarine, mayonnaise^b^	0.0 (0.4)	0.0 (0.8)	1.0^d^ (4.4)	0.60	76	—	—
Olive oil	3.8 (6.3)	10.0 (14.7)	6.9^d^ (10.0)	0.28	49	62	2
Rapeseed oil, walnut oil, mixed oil^b^	0.3 (2.0)	0.7 (2.7)	0.9^d^ (4.5)	0.35	47	—	—
Sunflower oil, groundnut oil^b^	0.0 (0.0)	0.0 (0.7)	0.5 (2.8)	0.21	65	—	—
Salty snacks^b^	0.0 (8.3)	4.0 (8.0)	-0.1 (7.5)	0.41	43	—	—
Sweet snacks, chocolate, and Danish pastries^b^	46.7 (50.4)	13.3 (40.0)	-26.6^d^ (61.0)	0.26	39	—	—
Fruit	202.8 (220.7)	351.0 (260.0)	122.3^d^ (274.7)	0.67	61	77	1
Water	992.8 (746.7)	1550.0 (1200.0)	1134.5^d^ (2410.6)	0.52	52	73	1
Coffee^b^	214.2 (303.3)	200.0 (380.0)	74.7 (303.4)	0.81	72	—	—
Tea and herb teas^b^	135.0 (557.1)	90.0 (400.0)	-3.1 (310.4)	0.88	71	—	—
Fruit juice^b^	50.0 (143.3)	50.0 (206.7)	29.8^d^ (132.6)	0.61	58	—	—
Sweet beverages^b^	0.0 (2.1)	0.0 (0.0)	-11.5^d^ (50.1)	0.73	79	—	—
Artificially-sweetened beverages^b^	0.0 (0.0)	0.0 (0.0)	10.8 (66.7)	0.55	88	—	—
Wine^b^	25.0 (90.0)	11.0 (40.5)	-16.4^d^ (68.7)	0.81	67	—	—
Alcoholic beverages excluding wine^b^	13.3 (52.5)	2.5 (20.0)	-24.3^d^ (65.5)	0.48	54	—	—

^a^Measured in grams (food) or milliliters (beverages).

^b^These food groups have a large proportion of non-consumers (>20%).

^c^Dashes indicate food groups that have a large proportion of nonconsumers (>20%). Classification into quintiles of consumption was not performed.

^d^The mean rank of the values of the three to six 24-hour recalls was significantly different to the mean rank of the values of the SFFeQ, according to Wilcoxon signed rank tests.

^e^Unadjusted Spearman correlation coefficients for which the statistical tests did not provide *P* values <.05.

The median proportion of participants classified in the same or adjacent quintiles of food group consumption by the FFeQ2, as well as by the mean of the 24-hour recalls, was 73%. The median proportion of participants classified in opposite quintiles was 1%. The median proportion of participants classified in the same tertile was 54%.

Mean macronutrient intakes estimated using the FFeQ2 did not differ in our study from those estimated in the 24-hour recalls (Table 3). Calcium and retinol intakes tended to be overestimated by the FFeQ (median 1113.7 mg/d, IQR 625.1 vs median 853.4 mg/d, IQR 322.5 and median 383.3 mg/d, IQR 393.4 vs median 0.0 μg/d, IQR 0.0, respectively) whereas alcohol and sodium intakes were underestimated by the FFeQ (median 2.0 g/d, IQR 6.9 vs median 4.7 mg/d, IQR 14.0 and median 2376.7 mg/d, IQR 945.7 vs median 2463.5 μg/d, IQR 1072.0, respectively).

Unadjusted correlation coefficients ranged from 0.08 (manganese and copper) to 0.77 (alcohol), with a 0.41 median value. Deattenuation mainly improved energy-adjusted correlation coefficients. Deattenuated energy-adjusted CC ranged from 0.05 (manganese) to 0.68 (potassium, carotene and vitamin C), with a 0.50 median value. A total of eight nutrients (sodium, magnesium, manganese, iron, copper, zinc, iodine and vitamin B12) had correlation coefficients lower than 0.3.

The median of percentages of participants classified in the same or adjacent quintiles of nutrient intakes by FFeQ2, and by the mean of the 24-hour recalls, was 66%. The median proportion of participants classified in opposite quintiles was 3%.

**Figure 1:**
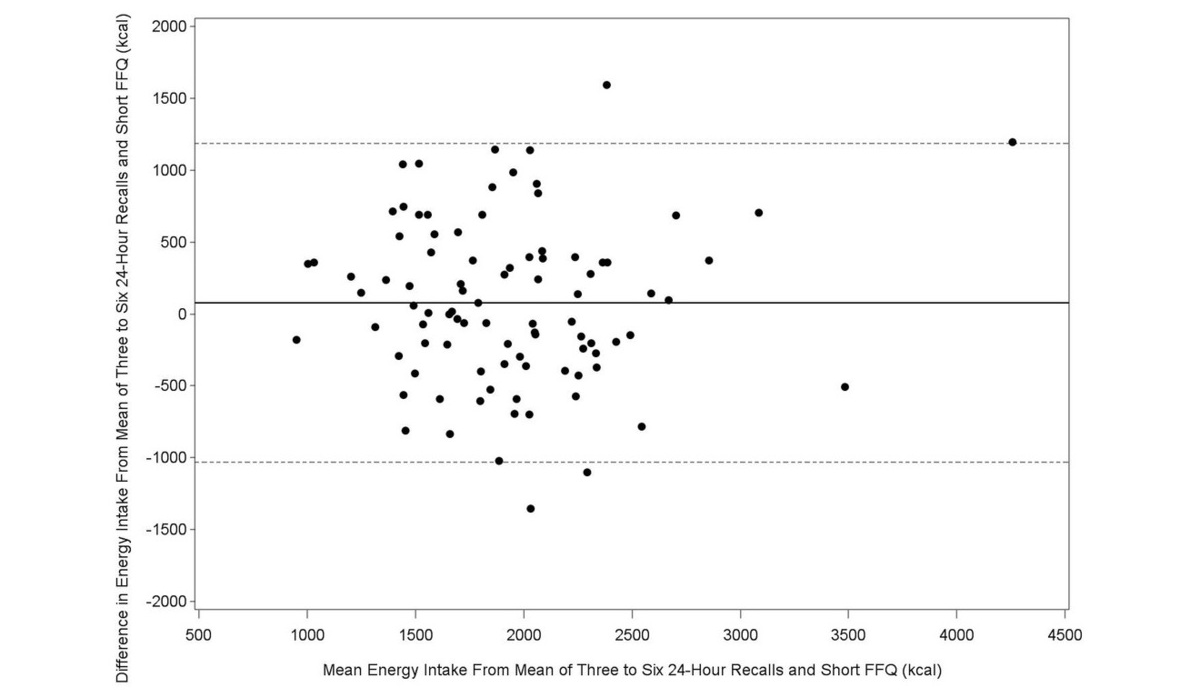
Bland-Altman plots related to energy. Difference in the daily intake of energy (crude variable) derived from the three to six 24-hour recalls and the short food frequency e-questionnaire (SFFeQ2) plotted against the corresponding mean daily intakes derived from the two methods. Solid lines represent mean difference, and dashed lines show lower and upper 95% limits of agreement (mean, SD 1.96; n=92).

**Figure 2:**
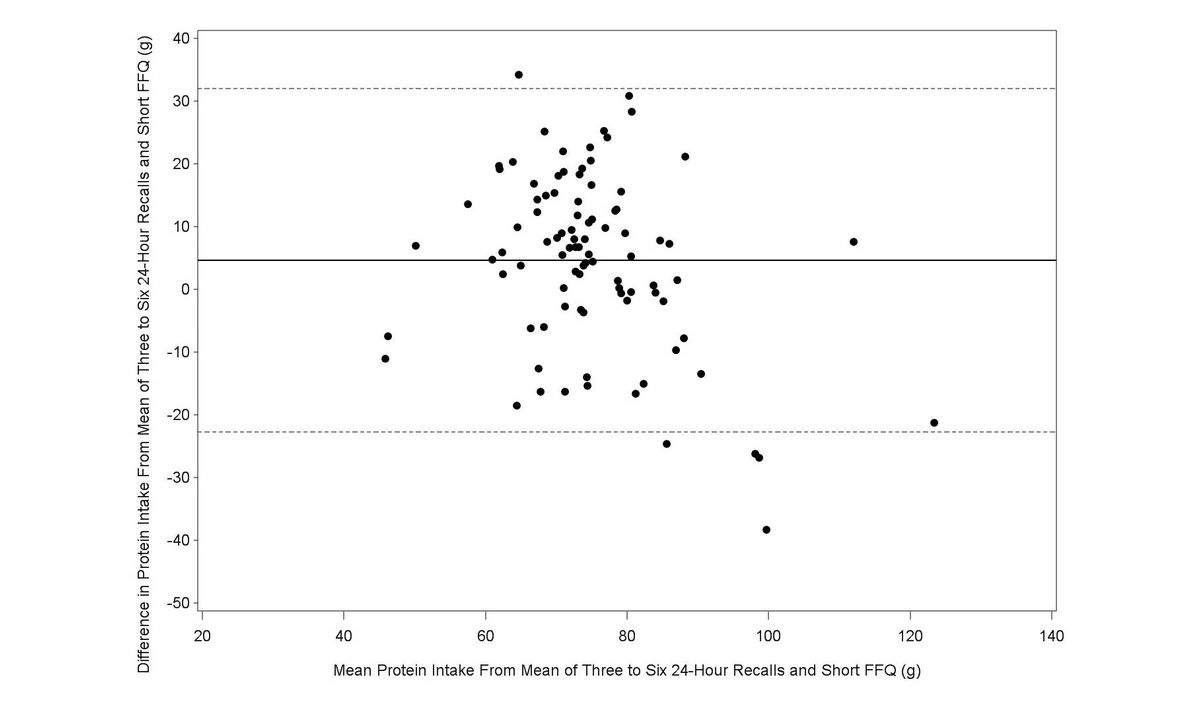
Bland-Altman plots related to protein. Difference in the daily intake of energy-adjusted protein derived from the three to six 24-hour recalls and the short food frequency e-questionnaire (SFFeQ2) plotted against the corresponding mean energy-adjusted daily intakes derived from the two methods. Solid lines represent mean difference, and dashed lines show lower and upper 95% limits of agreement (mean ± 1.96 SD) (n=92).

**Figure 3:**
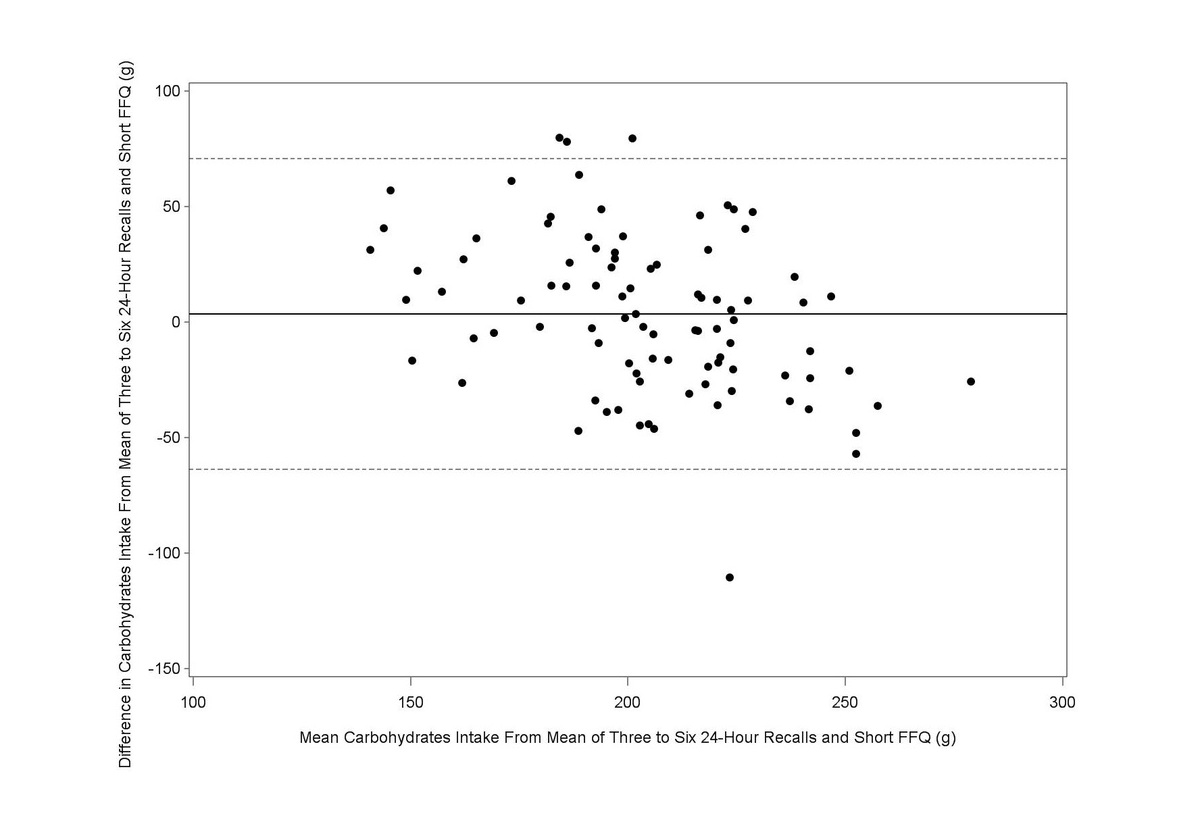
Bland-Altman plots related to carbohydrate. Difference in the daily intake of energy-adjusted carbohydrate derived from the three to six 24-hour recalls and the short food frequency e-questionnaire (SFFeQ2) plotted against the corresponding mean energy-adjusted daily intakes derived from the two methods. Solid lines represent mean difference, and dashed lines show lower and upper 95% limits of agreement (mean, SD 1.96; n=92).

**Figure 4:**
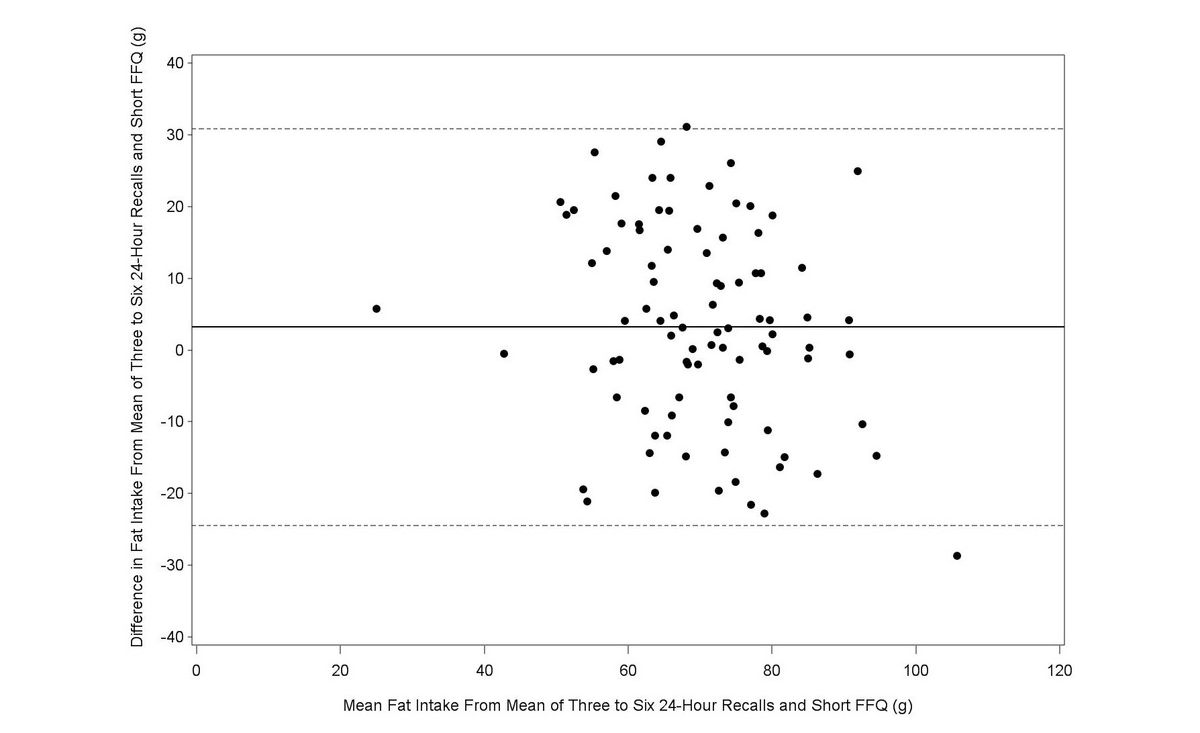
Bland-Altman plots related to lipid. Difference in the daily intake of energy-adjusted lipid derived from the three to six 24-hour recalls and the short food frequency e-questionnaire (SFFeQ2) plotted against the corresponding mean energy-adjusted daily intakes derived from the two methods. Solid lines represent mean difference, and dashed lines show lower and upper 95% limits of agreement (mean, SD 1.96; n=92).

**Figure 5:**
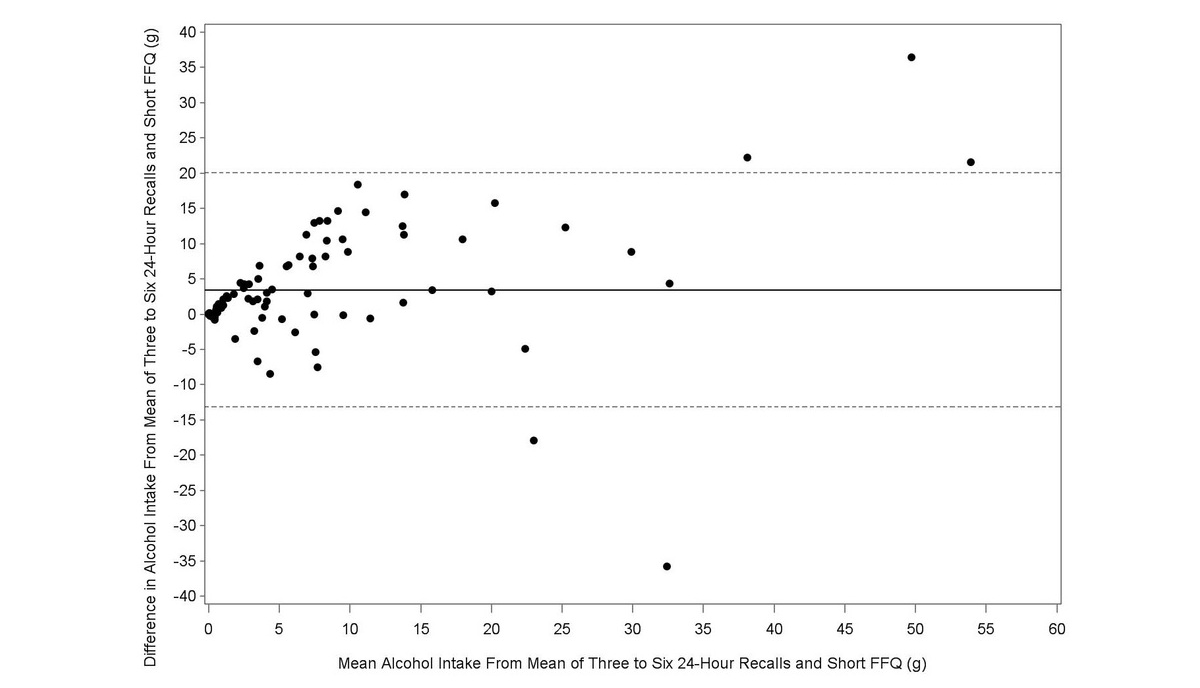
Bland-Altman plots related to alcohol. Difference in the daily intake of alcohol (crude variable) derived from the three to six 24-hour recalls and the short food frequency e-questionnaire (SFFeQ2) plotted against the corresponding mean daily intakes derived from the two methods. Solid lines represent mean difference, and dashed lines show lower and upper 95% limits of agreement (mean, SD 1.96; n=92).

**Figure 6:**
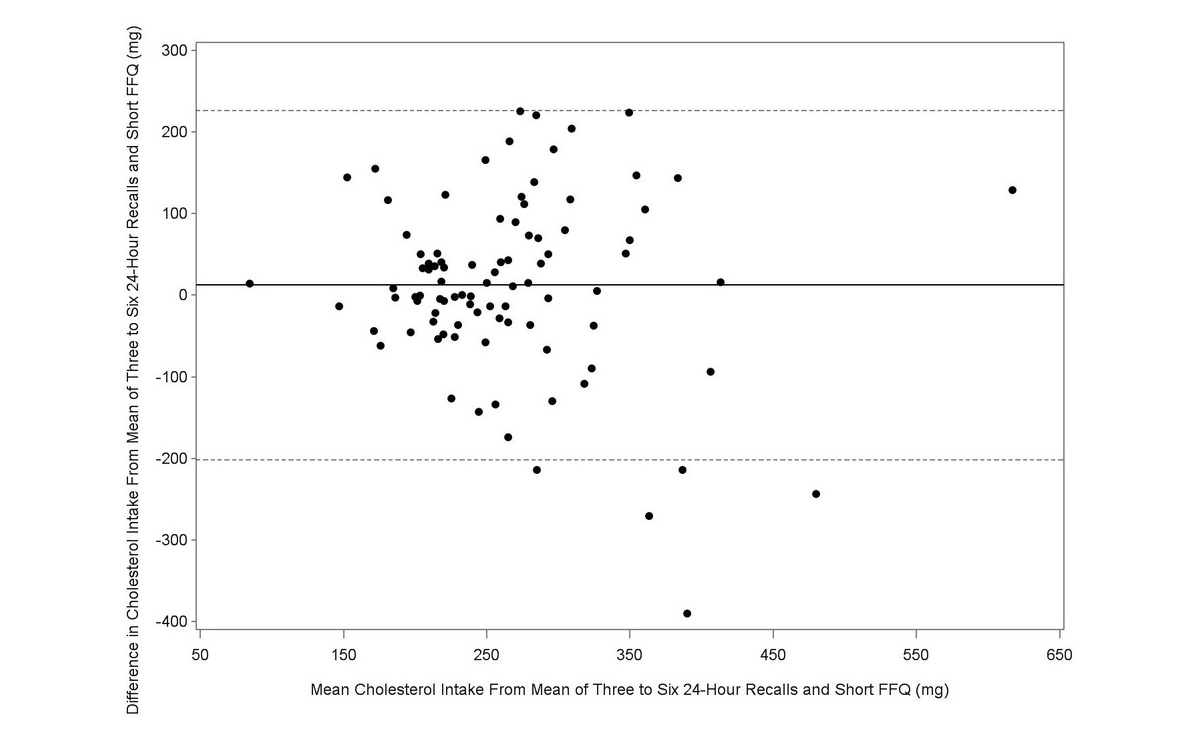
Bland-Altman plots related to cholesterol. Difference in the daily intake of energy-adjusted cholesterol derived from the three to six 24-hour recalls and the short food frequency e-questionnaire (SFFeQ2) plotted against the corresponding mean energy-adjusted daily intakes derived from the two methods. Solid lines represent mean difference, and dashed lines show lower and upper 95% limits of agreement (mean, SD 1.96; n=92).

**Figure 7:**
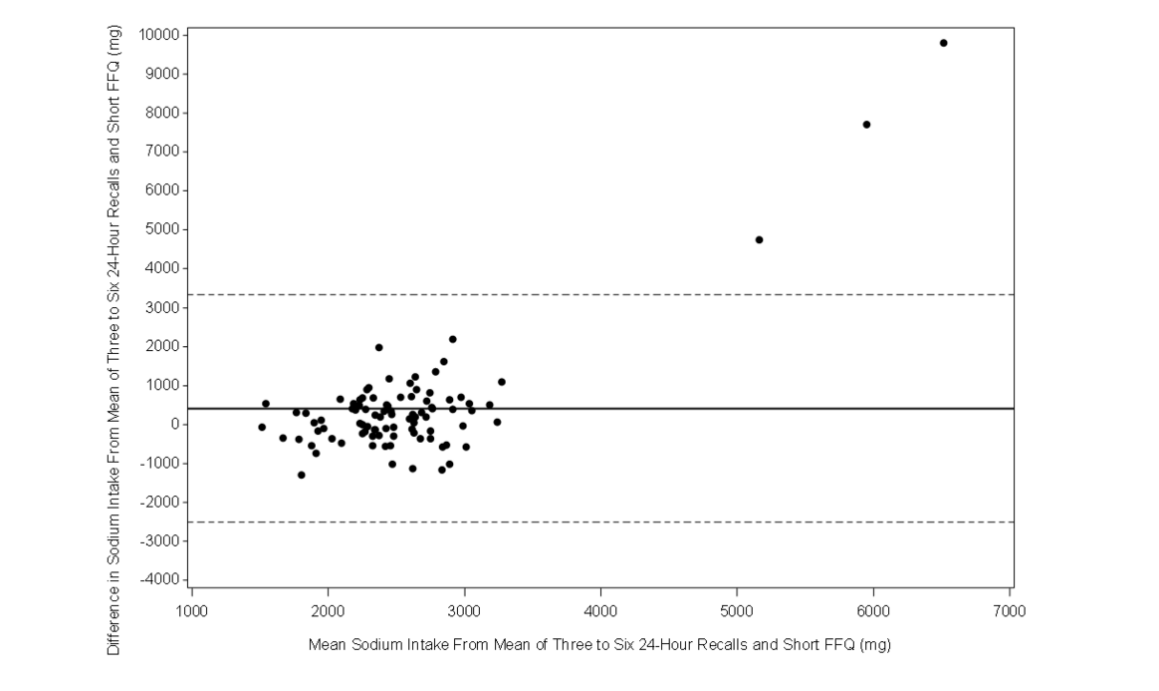
Bland-Altman plots related to sodium. Difference in the daily intake of energy-adjusted sodium derived from the three to six 24-hour recalls and the short food frequency e-questionnaire (SFFeQ2) plotted against the corresponding mean energy-adjusted daily intakes derived from the two methods. Solid lines represent mean difference, and dashed lines show lower and upper 95% limits of agreement (mean, SD 1.96; n=92).

**Figure 8:**
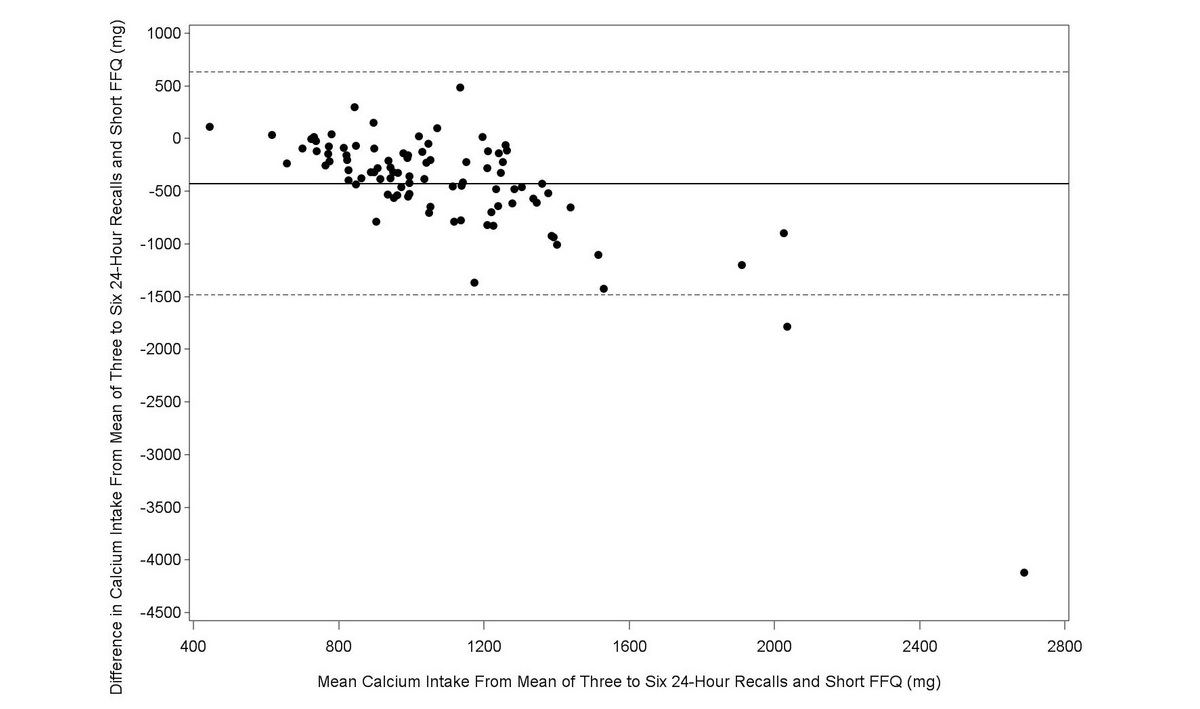
Bland-Altman plots related to calcium. Difference in the daily intake of energy-adjusted calcium derived from the three to six 24-hour recalls and the short food frequency e-questionnaire (SFFeQ2) plotted against the corresponding mean energy-adjusted daily intakes derived from the two methods. Solid lines represent mean difference, and dashed lines show lower and upper 95% limits of agreement (mean, SD 1.96; n=92).

The Bland-Altman plot analysis graphs displayed good agreement between the two methods of estimation across the range of intake for energy (Figure 1), protein (Figure 2), carbohydrates (Figure 3), lipids (Figure 4), alcohol (Figure 5), cholesterol (Figure 6), sodium (Figure 7), and calcium intakes (Figure 8). For all 33 studied nutrients, the mean difference between methods (FFeQ2 vs means of 24-hour recalls) was close to zero for all levels of intake, except for calcium (data not shown). Across the range of intakes, calcium was systematically overestimated by the FFeQ2 which was consistent with the results displayed in Table 3. The percentage of points that were outside the limits of agreement ranged from 1.1% (zinc and iodine) to 7.6% (sugars, and vitamins D, B1, and B6), with a median value of 4.3%, which, theoretically is the percentage of values outside the mean (SD 1.96). Finally, the agreement did not differ between subjects with high and low intakes.

**Table 3:** Relative validity of the FFeQ for nutrients (n=92). Means and cross-classification were computed on crudes variables. All variables were log transformed before computing Pearson correlation coefficients to improve normality.

Nutrients	Daily intakes			FFeQ2 vs mean of three to six 24-hour recalls				
	24-hour recalls, median (IQR)	FFeQ2, median (IQR)	FFeQ2-24-hour recalls, mean (SD)	Pearson correlation coefficients^a^			Cross-classification of nutrient distribution, %	
				Unadjusted	Energy-adjusted^b^	Deattenuated^c^	Subjects classified in same or adjacent quintile	Subjects classified in opposite quintiles
Energy (kcal)	1882.2 (659.6)	1859.5 (819.8)	-77.4 (565.3)	0.47	N/A^d^	0.50^e^	66	3
Protein (g)	76.2 (22.6)	72.2 (34.1)	-3.5 (23.6)	0.57	0.47	0.52	72	2
Carbohydrates (g)	203.7 (74.6)	202.3 (98.0)	-1.3 (72.6)	0.44	0.49	0.54	71	3
Fat (g)	74.2 (28.4)	67.7 (34.8)	-3.6 (27.2)	0.47	0.55	0.61	59	3
SFA^f^ (g)	26.9 (13.9)	25.8 (14.2)	-2.4^g^ (10.5)	0.61	0.54	0.61	67	1
MUFA^h^ (g)	24.2 (10.4)	26.3 (12.0)	3.1^g^ (12.2)	0.35	0.46	0.53	58	7
PUFA^i^ (g)	8.6 (3.7)	7.8 (3.6)	-0.1 (4.5)	0.32	0.48	0.55	58	4
Cholesterol (mg)	244.2 (133.6)	243.3 (130.2)	-11.4 (149.9)	0.46	0.31	0.39	65	5
Sugars (g)	79.8 (34.3)	82.5 (46.2)	3.6 (42.9)	0.28	0.39	0.45	71	8
Fiber (g)	21.6 (9.0)	20.9 (9.3)	-0.7 (7.9)	0.40	0.52	0.60	64	3
Alcohol (g)	4.7 (14.0)	2.0 (6.9)	-3.5^g^ (8.5)	0.77^j^	—^k^	—^k^	88	1
Water (g)	2736.5 (868.5)	3182.4 (1744.0)	1310.8^g^ (2654.0)	0.47	0.47	0.51	68	1
Sodium (mg)	2463.5 (1072.0)	2376.7 (945.7)	-402.5^g^ (1483.9)	0.37	*0.05*	0.07	64	1
Magnesium (mg)	319.1 (104.6)	344.0 (194.9)	52.0^g^ (142.4)	0.26	0.25	0.29	62	8
Phosphorus (mg)	1139.6 (334.3)	1114.7 (491.7)	1.6^g^ (352.8)	0.50	0.40	0.45	67	2
Potassium (mg)	3099.1 (1044.0)	3091.6 (1555.0)	133.1 (955.3)	0.49	0.59	0.68	71	2
Calcium (mg)	853.4 (322.5)	1113.7 (625.1)	442.2^g^ (553.1)	0.42	0.38	0.44	70	5
Manganese (mg)	2.8 (1.5)	11.5 (6.5)	9.3^g^ (4.9)	*0.08*	*0.05*	0.05	59	8
Iron (mg)	9.4 (4.3)	10.6 (5.0)	1.1^g^ (4.5)	0.28	0.23	0.27	62	3
Copper (mg)	1.4 (0.6)	2.2 (1.5)	0.8^g^ (1.6)	*0.08*	*0.06*	0.07	57	7
Zinc (mg)	8.6 (2.9)	9.1 (4.7)	0.4 (5.0)	0.40	0.22	0.26	61	1
Iodine (μg)	120.2 (68.5)	122.1 (57.2)	-3.6 (69.1)	0.43	0.24	0.29	68	2
Retinol (μg)	0.0 (0.0)	383.3 (393.4)	506.6^g^ (479.8)	0.11^j^	—^k^	—^k^	41	18
Carotene (μg)	3298.9 (2559.0)	3165.6 (2944.0)	-306.4 (2833.2)	0.52	0.54	0.68	77	3
Vitamin D (μg)	2.2 (1.5)	2.1 (1.2)	-0.4 (1.9)	0.32	0.23	0.31	61	3	
Vitamin E (mg)	9.0 (4.3)	10.2 (5.1)	2.1^g^ (5.4)	0.25	0.43	0.53	58	4
Vitamin C (mg)	118.6 (69.7)	124.7 (90.3)	13.3 (68.5)	0.54	0.56	0.68	78	2
Vitamin B1 (mg)	1.1 (0.4)	1.0 (0.4)	-0.1^g^ (0.4)	0.31	0.33	0.41	66	4
Vitamin B2 (mg)	1.6 (0.6)	1.5 (0.8)	0.0 (0.6)	0.45	0.48	0.53	68	1
Vitamin B3 (mg)	16.5 (6.8)	15.6 (8.9)	0.0 (6.7)	0.49	0.49	0.58	73	4
Vitamin B5 (mg)	4.8 (2.0)	4.8 (2.4)	0.2 (1.8)	0.47	0.57	0.64	70	1
Vitamin B6 (mg)	1.7 (0.7)	1.6 (0.6)	-0.1 (0.7)	0.28	0.38	0.46	58	2	
Vitamin B9 (μg)	305.1 (115.1)	321.0 (141.0)	26.9 (126.0)	0.36	0.49	0.58	64	1
Vitamin B12 (μg)	3.3 (2.3)	5.6 (3.8)	2.1^g^ (6.0)	0.31	0.21	0.26	65	7

^a^The statistical tests provided *P* values <.05 for each Pearson correlation coefficient, except for those in italics.

^b^Energy adjustment according to the residual method.

^c^Energy-adjusted and deattenuated Pearson correlation coefficient (corrected for within-person variation in the three to six 24-hour recalls).

^d^N/A: not applicable

^e^Unadjusted and de-attenuated Pearson correlation coefficient.

^f^SFA: saturated fatty acids.

^g^The mean rank of the values of the three to six 24-hr recalls was significantly different to the mean rank of the values of the SFFeQ, according to Wilcoxon signed rank tests. The statistical tests provided *P* values <.05 for each Pearson correlation coefficient, except for those in italics.

^h^MUFA: monounsaturated fatty acids.

^i^PUFA: polyunsaturated fatty acids.

^j^Spearman correlation coefficients were performed because normality was not respected.

^k^Normality was not respected. Energy-adjusted and deattenuated coefficients were not performed.

### Reproducibility

Absolute daily intakes of food groups were mostly comparable between the two FFeQs (see Multimedia Appendix 5). A rather large statistically significant decrease was observed for the “sweet beverages” and “artificially-sweetened beverages” between FFeQ1 and FFeQ2.

Unadjusted Spearman correlation coefficients ranged from 0.34 (sunflower and groundnut oils) to 0.90 (wine), with the median value being 0.65. Intraclass correlation coefficients ranged from 0.33 (sweet snacks, chocolate, and Danish pastries) to 0.72 (poultry or rabbit, fish, and fruit), with the median value being 0.60.

The median of percentages of subjects classified in the same or adjacent quintiles of food group consumption by both FFeQs was 80%. The median proportion of participants classified in opposite quintiles was 1%. The median percentage of subjects classified in the same tertile was 64%.

Absolute daily intake of energy and nutrients were comparable between the two FFeQs, although all nutrient intakes (excluding alcohol) showed a slight but statistically significant decrease between FFeQ1 and FFeQ2 (see Multimedia Appendix 6).

Crude correlation coefficients ranged from 0.58 (iron) to 0.89 (alcohol), with a 0.65 median value. Energy-adjusted Pearson correlation coefficients ranged from 0.54 (vitamin B1) to 0.77 (vitamin E), with a 0.65 median value. Intraclass correlation coefficients ranged from 0.55 (carbohydrates) to 0.73 (magnesium and manganese), the median value being 0.65.

The median proportion of subjects classified in the same or adjacent quintiles of nutrient intakes by both FFeQs was 79%. The median proportion of participants classified in opposite quintiles was 1%.

## Discussion

### Principal Findings

The present study investigated the relative validity and reproducibility of a new FFeQ, in a sample of French adults. The FFeQ was designed to accurately estimate energy intake and rank participants according to their dietary and nutrient intakes. The overall results indicate acceptable relative validity (for nutrient intakes, median correlation coefficient=0.50 and median proportion of subjects classified in the same or adjacent quintiles by the FFeQ2 and the 24-hour recalls=66%), and good reproducibility (for nutrient intakes, median correlation coefficient=0.65, and median proportion of subjects classified in the same or adjacent quintiles=79%). Our tool demonstrated an acceptable ability to rank participants for most nutrients and food groups, making it sufficiently informative when studying associations with health outcomes and when adjusting for nutritional intake in epidemiological and clinical studies [6,42]. It can also be used to derive dietary patterns using collected food data.

Because combinations of different assessment methods are becoming increasingly popular and can address several methodological limitations [43], it would be interesting, in further analyses to study under which circumstances a combination of the FFeQ with dietary recalls would be more efficient than either the FFeQ or the dietary recalls alone to address precision, power, and sample size, as it has been previously done [44].

Our FFeQ was composed of 50 items, of which 44 were used to obtain nutritional data. In their review, Cade et al reported that the number of items in FFQs published between 1980 and 1999 ranged from three to 350 items [45], with the median being 79 items. However, according to Willett [6], there is a rapidly decreasing marginal gain in information obtained with increasingly detailed questionnaires. Considering the relative validity and reproducibility of our FFeQ, it appears that the chosen 44 items were sufficient to assess the overall diet and to describe major food and nutrient intakes. The remaining six items of the online FFeQ will help provide meaningful insights as they could be used for qualitative studies or to stratify statistical analyses according to eating habits like meal frequency, socialization during meals or source of food supplies.

### Relative Validity

Recovery biomarkers are considered gold-standard measures to validate self-reported intakes. However, because of their costliness and because only a few recovery biomarkers are currently known, they are rarely used in validity studies [43]. When selecting a reference method to validate a tool, errors of both methods must be as independent as possible [46]. Even if correlated errors related to memory, perception of serving sizes and social desirability exist between FFQs and 24-hour recalls, multiple 24-hour recalls have often been considered as the best feasible reference method [47]. Here, to study the FFeQ relative validity, three to six 24-hour recalls were used as the reference method.

Our study showed acceptable relative validity for food (correlation coefficients median and range: 0.51 [0.09-0.88]) and nutrients (correlation coefficients median and range: 0.40 [0.05-0.68]), and our results were comparable to those from other studies [14,21,32,33,46,48-52]. In the NIH–AARP study, validity coefficients for energy-adjusted nutrients ranged from 0.36 to 0.76 [46].

For validation studies, it has been suggested that correlation coefficients should be ≥ 0.3, preferably over 0.4, and optimally in the range of 0.5-0.7 [6,45,53]. In our study, only eight food groups out of 40 and eight nutrients out of 34 had correlation coefficients lower than 0.3.

For food groups, the lowest correlation coefficients were found for foods that are not consumed regularly such as “variety meats,” “whole-grain pasta, rice and wheat,” “legumes,” and “sunflower oil and groundnut oil”. Such findings on rarely consumed foods have been previously reported [32-34]. Low correlation coefficients were also observed for mixed items such as the “sweet snacks, chocolate, and Danish pastries” item. Due to the number of foods included in these groups, consumption may be difficult to estimate. Finally, a low correlation coefficient was observed for the “potatoes and other tubers” item. The estimation of its consumption may have been difficult due to the large consumption of mixed dishes including potatoes in France. Underestimation of water during the 24-hour recall has previously been reported [34] arguing that even if its consumption is systematically asked, it is easily forgotten.

Because of social desirability [42,54], food groups such as “alcoholic beverages” and “fruit” may have been under- and overestimated respectively. Over-reporting of fruit and vegetable intake by subjects seeking social approval is a common bias [55]. As previously reported [56,57], correlation coefficients were lower for vegetables than for fruit. According to Wakai [57], it may be partly because the frequency of fruit consumption is easier to report than vegetables because fruit is more often consumed raw whereas vegetables are more frequently part of cooked dishes and therefore not integrally recalled. Furthermore, fruit is frequently consumed as a single food item and comes in natural or typical units, whereas vegetables are often sliced or cut which makes them more difficult to quantify [56].

In our study, unadjusted correlation coefficients for macronutrients ranged from 0.47 to 0.57. Similar results were previously obtained for online FFQs (range: 0.06-0.68 [21,48,50], FFQs of 50 items max; range: 0.22-0.53 [14,51,52] and FFQs developed for adults; range: 0.29-0.61 [32,33,49]).

All macronutrients had correlation coefficients in the range of 0.5-0.7. Regarding the nutrients, the highest correlation coefficient was observed for alcohol (unadjusted correlation coefficient of 0.77). One of the lowest coefficients was observed for sodium (deattenuated energy adjusted correlation coefficient of 0.07). As reported in the pilot study (validation study of the paper version of the FFQ among patients with chronic kidney disease [8]), even though a question about salt added after food preparation was asked in the specific part of the questionnaire, it was still difficult to estimate its intake. However, when looking at individuals’ rankings, 64% of participants were classified in the same or adjacent quintile in terms of sodium intake when comparing the FFeQ2 and the 24-hour recalls.

After adjustment for energy some correlation coefficients were increased, and others were decreased. According to Willett et al [58], energy adjustment can increase the correlation coefficients when the variability of the nutrient intake is related to energy intake, or it can decrease when the variability of the nutrient is subject to systematic errors of under or overestimation of reported food consumption.

Despite some differences in estimations in both foods and nutrients by the questionnaire, agreement in classification was comparable to what other studies have shown [59] or slightly lower than shown in other studies [14,32]. Our results were close to the recommended 70% [60]. The highest level of participants classified in opposite quintiles was observed for retinol (18%). One of the main sources of retinol are variety meats, which were rarely consumed and for which consumption was probably difficult to evaluate with only three to six 24-hour recalls.

### Reproducibility

Our study showed acceptable reproducibility for most foods (ICC range: 0.33-0.72, median 0.60) and nutrients (ICC range: 0.55-0.73, median 0.65). Our findings were comparable to prior reported correlation coefficients for reproducibility [25,49,61]. An important factor influencing reproducibility is the period between the two questionnaires. We adopted a one-year time interval which is long but frequently used and reported as acceptable [45,62,63]. However, we cannot exclude that some dietary changes may have occurred during the period. The reproducibility observed here may therefore be lower than the true value.

As previously reported in the literature, a slight decrease in food and nutrient intakes was observed between FFeQ1 and FFeQ2 [16,25,32,64,65]. Due to the completion of the 24-hour recalls and the FFeQ1, a learning effect may explain this trend [66]. In favor of this hypothesis, several authors found that the second FFQ, which indicated reduced nutrient intake, was more valid than the first one when compared to 24-hour recalls [32,65,67].

According to a review, correlation coefficients of 0.5 to 0.7 between two administrations are commonly reported [45]. In our study, 75% and 100% of the studied food groups and nutrients had correlation coefficients ≥0.5. Not one food group had a correlation coefficient lower than 0.3.

Agreement in classification was very good (median of 80% and 79% for food and nutrient intakes respectively). For all nutrient intakes 74% to 94% of participants were classified in the same or adjacent quintile.

### Strengths and Limitations

The current work has some limitations. People involved in the current study were volunteers. Volunteers may be more health-conscious, pay more attention to their diets than the average population, and therefore provide more accurate responses to questionnaires. However, subjects participating in observational epidemiological or clinical studies that are likely to use this tool in the future are also volunteers.

Our work has several strengths. The tool we developed was easy to complete and not time consuming. The implementation of photographs helped the participants estimate the amounts of food consumed and it has previously been shown that the use of photographs improves the ability to report the true quantity of dietary intakes [68].

In addition, a total of 92 participants were included in our study. It is higher than the reported number in recent studies [21,69,70].

Here, we present relative validity results for the online FFQ in a sample of French adults. A paper version of the FFQ has previously been validated in a sample of patients with chronic kidney disease [8] and further validity studies will now be conducted in specific population subgroups (for example, adolescents or cancer survivors). One of the main strengths of the consortium is that we will have a unique tool (due to the shared 40 items in the first part of the FFQ), that is useful for the comparison between several populations. The questionnaire is now available for other epidemiological and clinical studies interested in assessing the habitual diet quickly. We validated a Web-based version of the FFQ which provides valuable insights: it enables an interactive interface for participants and improves the quality of answers by directly including cutoff values and messages of alert in case of inconsistent, abnormal, or missing data.

### Conclusions

For most food groups and nutrients, the FFeQ showed acceptable relative validity and reproducibility in a sample of French adults. It appears to be valid to rank individuals based on their food and nutrient intakes and can now be used in large-scale epidemiological studies as well as in clinical routine to easily and quickly assess the habitual diet. Developing an evidence-based smartphone application from the FFeQ is the next step. This type of tool may further be used to monitor patients’ nutrient intakes and provide them with instantaneous feedback and nutritional recommendations about their diets.

## Appendix

Multimedia Appendix 1 Flow diagram.

Multimedia Appendix 2 Extract from the SFFeQ, French and original version.

Multimedia Appendix 3 Extract from the SFFeQ, version translated in English.

Multimedia Appendix 4 SFFQ items used to obtain nutritional data (n=44).

Multimedia Appendix 5 Reproducibility of food group consumptions of the SFFeQ (n=223).

Multimedia Appendix 6 Reproducibility of nutrient intakes of the SFFeQ (n=223).

## Abbreviations

BMI: body mass index
BMR: basal metabolic rate
FFQ: Food Frequency Questionnaire
FFeQ: Food Frequency e-Questionnaire
