# Supplementary figures and images for: Relative Validity and Reproducibility of a New 44-Item Diet and Food Frequency Questionnaire Among Adults: Online Assessment
Source: J Med Internet Res. 2018 Jul 5;20(7):e227. doi: 10.2196/jmir.9113 (PMC6053608; doi:10.2196/jmir.9113)

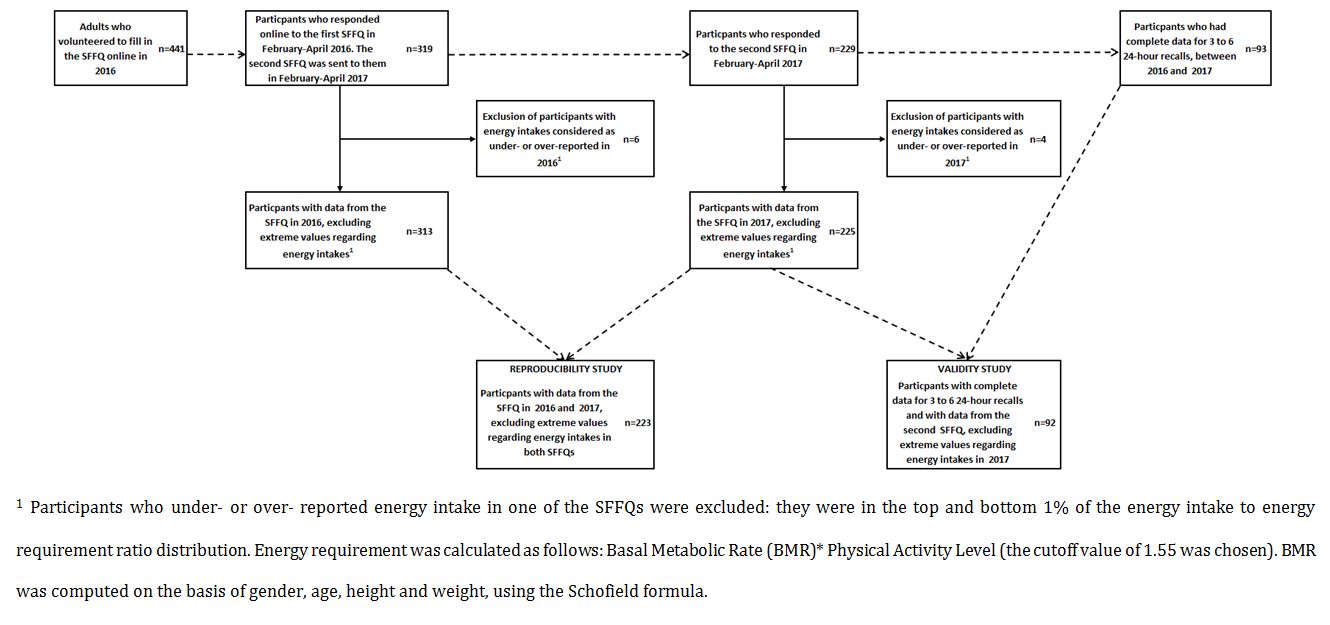

Supplement: Multimedia Appendix 1 [file jmir_v20i7e227_app1.JPG]

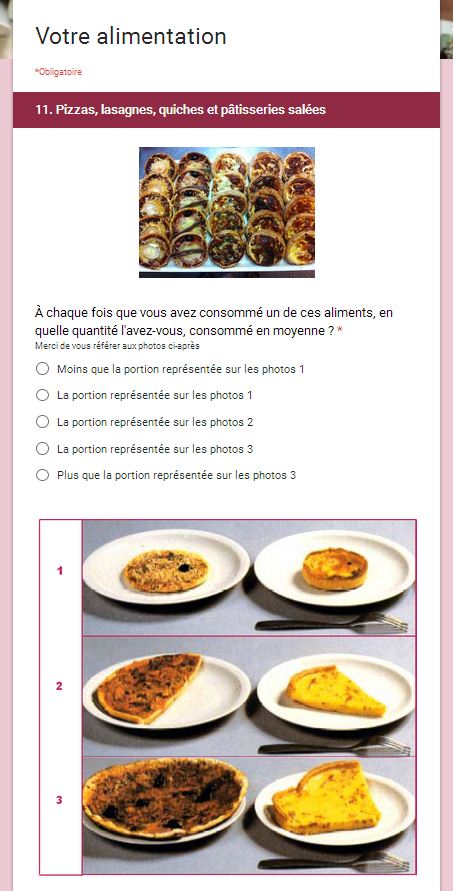

Supplement: Multimedia Appendix 2 [file jmir_v20i7e227_app2.jpg]

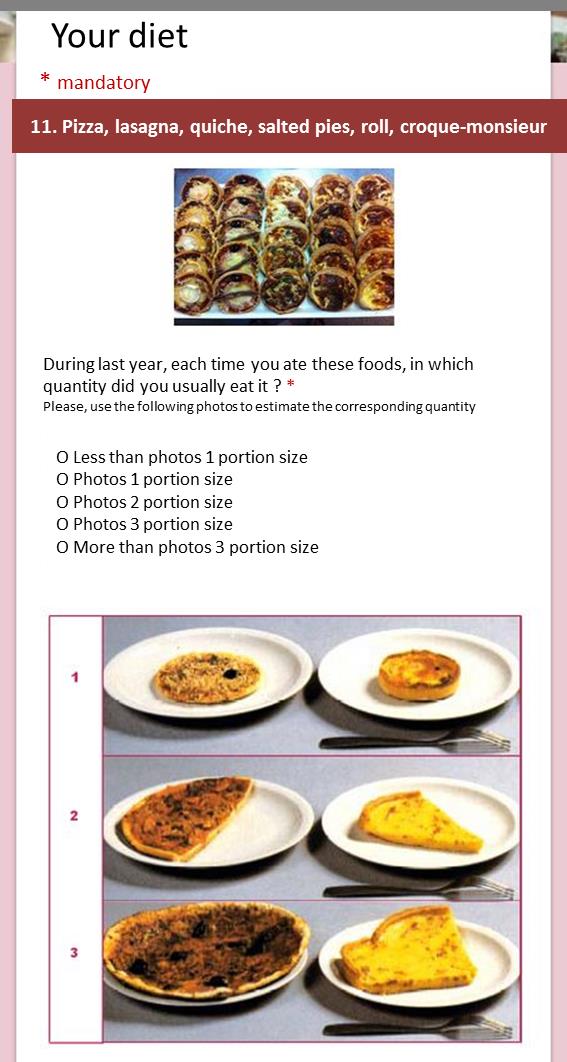

Supplement: Multimedia Appendix 3 [file jmir_v20i7e227_app3.jpg]

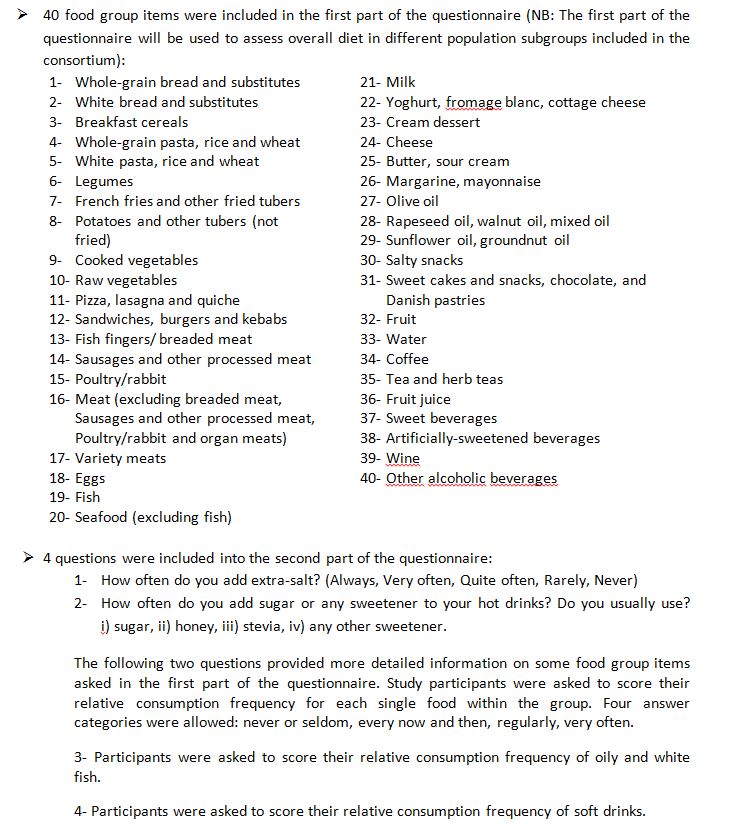

Supplement: Multimedia Appendix 4 [file jmir_v20i7e227_app4.JPG]

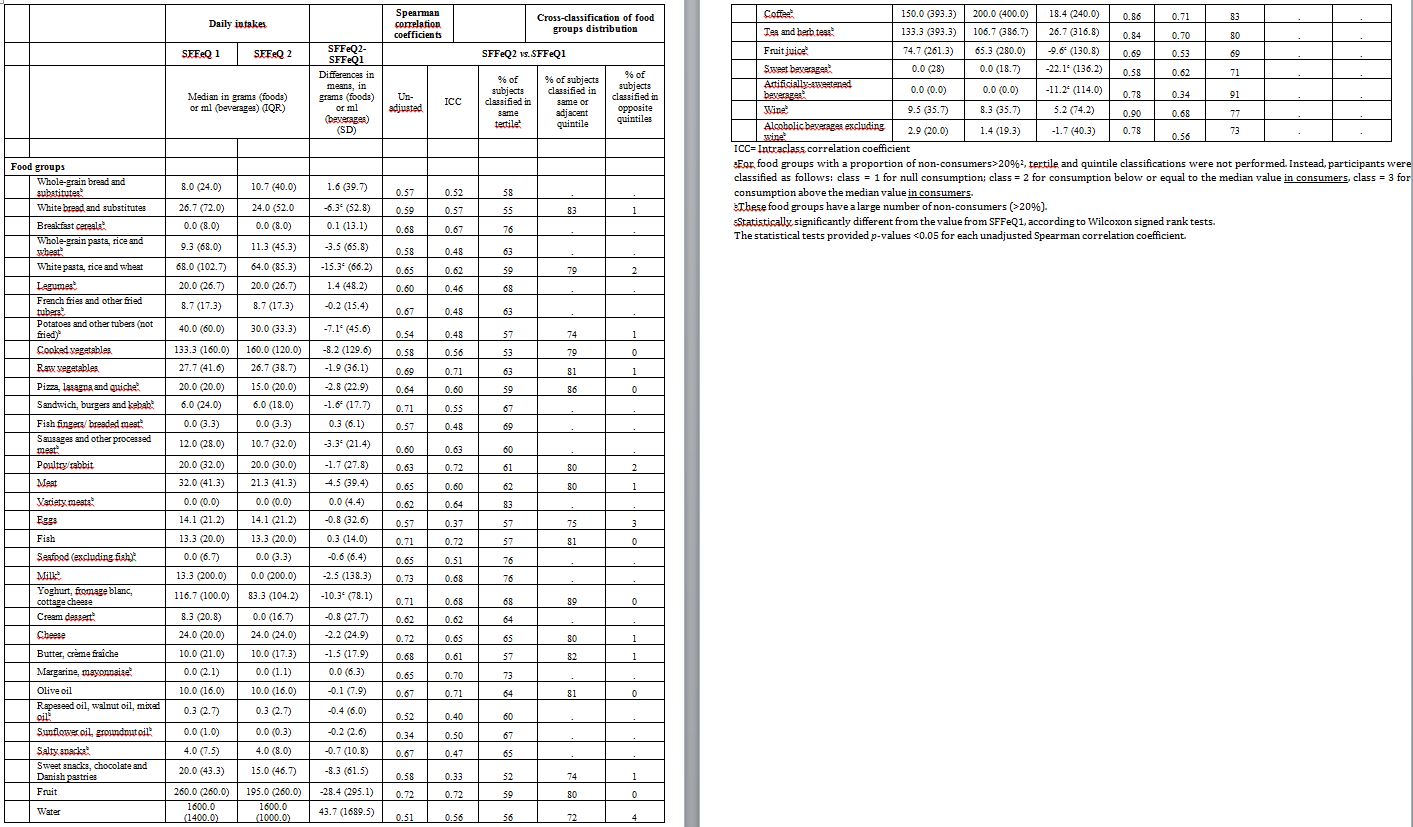

Supplement: Multimedia Appendix 5 [file jmir_v20i7e227_app5.JPG]

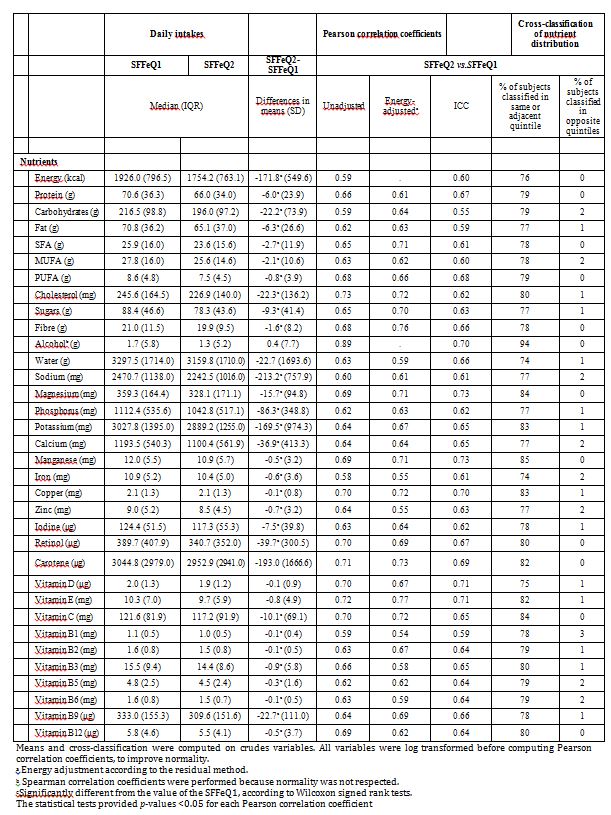

Supplement: Multimedia Appendix 6 [file jmir_v20i7e227_app6.JPG]
